# Adaptive behavioural syndromes due to strategic niche specialization

**DOI:** 10.1186/1472-6785-7-12

**Published:** 2007-10-12

**Authors:** Ralph Bergmüller, Michael Taborsky

**Affiliations:** 1 Department of Behavioural Ecology, Institute of Zoology, University of Bern, Wohlenstr. 50a, CH-3032 Hinterkappelen, Switzerland; 2 Department of Eco-Ethology, Institute of Biology, University of Neuchâtel, Rue Emile Argand 11, CH-2009 Neuchâtel, Switzerland

## Abstract

**Background:**

Behavioural syndromes, i.e. consistent individual differences in behaviours that are correlated across different functional contexts, are a challenge to evolutionary reasoning because individuals should adapt their behaviour to the requirements of each situation. Behavioural syndromes are often interpreted as a result of constraints resulting in limited plasticity and inflexible behaviour. Alternatively, they may be adaptive if correlated ecological or social challenges functionally integrate apparently independent behaviours. To test the latter hypothesis we repeatedly tested helpers in the cooperative breeder *Neolamprologus pulcher *for exploration and two types of helping behaviour. In case of adaptive behavioural syndromes we predicted a positive relationship between exploration and aggressive helping (territory defence) and a negative relationship between these behaviours and non-aggressive helping (territory maintenance).

**Results:**

As expected, helpers engaging more in territory defence were consistently more explorative and engaged less in territory maintenance, the latter only when dominant breeders were present. Contrary to our prediction, there was no negative relationship between exploration and territory maintenance.

**Conclusion:**

Our results suggest that the three behaviours we measured are part of behavioural syndromes. These may be adaptive, in that they reflect strategic specialization of helpers into one of two different life history strategies, namely (a) to stay and help in the home territory in order to inherit the breeding position or (b) to disperse early in order to breed independently.

## Background

Evolutionary theory predicts that animals will behave adaptively, which means that their behavioural phenotype should on average converge towards an optimum. Hence, variation in behaviour has typically been interpreted as random variation around an adaptive mean or as a reflection of environmental or intrinsic constraints. However, studies on animal behaviour at the level of individuals have suggested that the observed variation can itself be adaptive [1-3]. More recently, individual differences in behaviours have been found to sometimes correlate across functionally unrelated contexts, a phenomenon termed 'behavioural syndromes', 'animal personalities' or 'animal temperaments' [4-7].

Apart from humans, behavioural syndromes have, for instance, been reported in mammals, birds, lizards, amphibians, fish, molluscs and arthropods [8] suggesting they are common in animals and reflect individual differences in coping styles [9]. Although the question why individual differences should be consistent over time has received some theoretical attention [[10], and cited references], a conceptual framework to explain the functional significance of correlations between different behaviours (or cross context correlations of the same behaviour) is only gradually emerging [6,11].

Currently, two non-exclusive hypotheses seek to explain behavioural syndromes [12]. One hypothesis, which we refer to as the 'constraint hypothesis' [6,12], proposes that behaviours are correlated due to common regulatory mechanisms, so they may not be free to evolve independently. Under such conditions, a behavioural trait that is beneficial in one context (e.g. aggression in resource competition) may carry over into another context, where it can be detrimental (e.g. aggression during courtship) [6]. Behavioural syndromes due to constraints may be expected when the negative fitness consequences of carry over effects are relatively small, when evolutionary stability has not yet been reached or when decoupling of traits or phenotypic plasticity would be too costly [2,6,13].

A second hypothesis, which we refer to as the 'adaptive hypothesis', proposes that behavioural syndromes may be adaptive in that they functionally integrate several traits that work well together [12,14,15]. This may be the case when correlated ecological challenges favour combinations of traits that are in principle unrelated [16-18], for instance, when life history tradeoffs result in polymorphic populations with regard to traits that affect several behaviours [19,20].

Cooperatively breeding species are of particular interest with regard to behavioural syndromes because in such species helpers may have two alternative life-history options which may be broadly described as (a) 'stay', when individuals stay in the home territory and eventually inherit the territory by queuing for the breeding position [21] or (b) 'disperse', when individuals disperse to acquire a breeding position elsewhere [22]. As these two options involve different and specific challenges, they may correspond to within-species social niches or strategies, and should demand different sets of behavioural tendencies corresponding to those pathways. For instance, stayers may influence whether they are tolerated in the dominants' territory, by paying 'rent' in order to be allowed to stay [23]. Under such conditions, helpers should engage in helping behaviours such as brood care and territory maintenance, but they should engage less in aggressive behaviours like territory defence, as this might carry over to within group aggression and result in conflict escalation. As stayers usually will not attempt to disperse, they should not be very explorative. In contrast, dispersers should only invest as much in helping as is required for temporary group membership. Therefore, they should show little brood care and territory maintenance, but they should tend to be explorative, which would help to find a vacant territory or breeding position. As they should spend more time at the edge of the territory and in order to be able to defend an own territory soon, dispersers should also tend to be more aggressive than stayers. Adopting one of both strategies should therefore involve distinct social and ecological challenges.

We studied the cooperatively breeding Lake Tanganyika cichlid *Neolamprologus pulcher *to investigate a potential relationship between different behaviours that may be associated with the different life history options, to stay or to disperse. Helpers in *N. pulcher *participate in several cooperative tasks including territory defence and maintenance, and direct brood care [24]. Previous studies suggested that helpers differ in helping propensity [25-28], aggression [27], exploration and dispersal behaviour [29,30]. Here we ask whether these individual differences are consistent over time and whether there is a specific relationship between these behaviours. We tested helpers repeatedly for exploration behaviour, and for two types of helping behaviour that serve different social functions, i.e. territory maintenance (removing sand from the breeding shelter, i.e. 'digging') and territory defence (aggressive behaviour against a same sized conspecific intruder). In case stayers and dipersers show adaptive combinations of behavioural traits (i.e. coping strategies), behavioural syndromes should correspond to the challenges present when choosing one of the two life history options: stayers should tend to show high levels of territory maintenance but a small propensity to explore or engage in territory defence, whereas dispersers should display the opposite tendencies. In other words, we predict (1) a positive correlation between the individual tendency to engage in territory defence and exploration and (2) negative correlations between (a) territory maintenance and exploration and (b) territory maintenance and territory defence.

## Results

### (a) Consistency of behavioural traits

Except for overt attacks against a conspecific intruder, the frequency of the measured behaviours was consistent within individuals between the measurements. Individuals showed similar exploration tendencies between the tests (individual: *F*_11;22 _= 7.28; *P *< 0.001) and there was no difference between the three different tests (test: *F*_2;22 _= 0.86; *P *= 0.44). After removing the factor 'test' from the analysis the resulting repeatability coefficient for exploration is *r *= 0.68 (individual: *F*_11;24 _= 7.37; *P *< 0.001). Overt attacks were not consistent within individuals (individual: *F*_12;12 _= 1.82; *P *= 0.16) and there was no effect of the treatment (dominants present/not present) (treatment: *F*_1;12 _= 0.16; *P *= 0.70). Aggressive displays were consistent within individuals (individual: *F*_12;12 _= 3.04; *P *= 0.03) and individuals displayed more often aggressively towards intruders when dominants were absent than when they were present (treatment: *F*_1;12 _= 13.04; *P *= 0.003). The estimated repeatability of aggressive displays corrected for treatment is *r *= 0.51. Digging behaviour was consistent within individuals (individual: *F*_12;11 _= 4.02; *P *= 0.01) and individuals tended to dig more when dominants were absent than when they were present (treatment: *F*_1;12 _= 3.67; *P *= 0.08). The estimated repeatability for digging corrected for treatment is *r *= 0.60.

### (b) Correlations between functionally different behaviours

**Figure 1:**
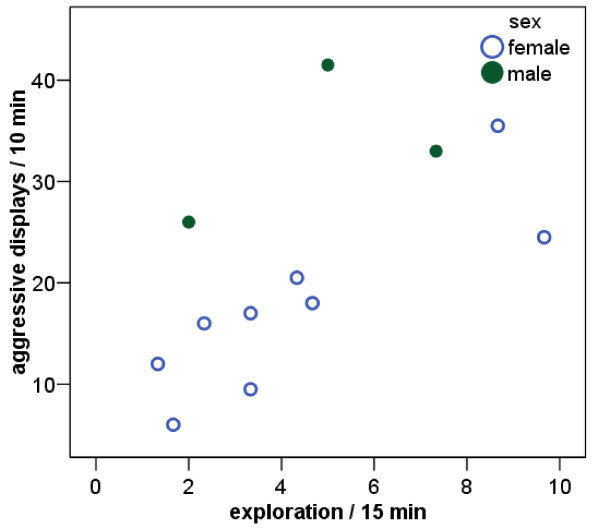
Exploration behaviour (the number of pot halves visited) was positively correlated with the frequency of aggressive displays towards an intruder (*r*_*s *_= 0.71, *N *= 12; *P *= 0.01). This relationship was also significant for females only (*r*_*s *_= 0.86, *N *= 9; *P *= 0.003).

**Figure 2:**
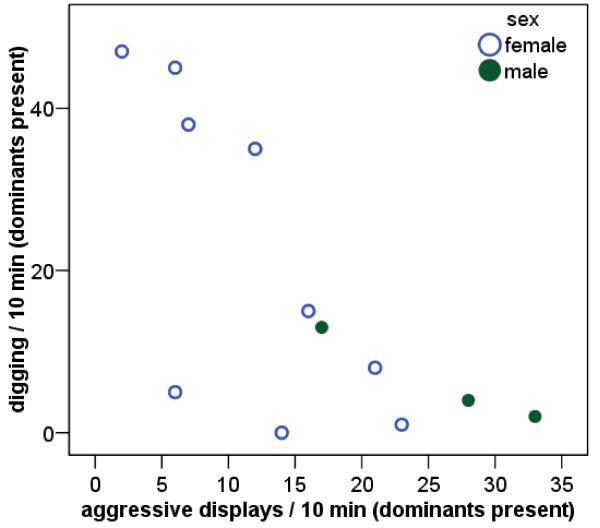
When breeders were present, the frequency of aggressive displays towards an intruder correlated negatively with the frequency of digging (*r*_*s *_= -0.69, *N *= 12; *P *= 0.01). This relationship was also significant for females only (*r*_*s *_= -0.66, *N *= 9, *P *= 0.05).

If behavioural syndromes exist according to strategic specializations into dispersers and stayers, we predicted (1) a positive correlation between exploration behaviour and territory defence and (2) a negative correlation between these behaviours and territory maintenance (digging). According to the first prediction, exploration behaviour correlated positively with aggressive displays against an intruder (Fig. 1). Contrary to the second prediction, digging behaviour was neither correlated with aggressive displays (Spearman rank correlation *r*_*s *_= -0.07, *N *= 12, *P *= 0.83) nor with exploration behaviour (*r*_*s *_= -0.02, *N *= 12, *P *= 0.95). However, when performing the analysis for each treatment (presence/absence of breeders) separately, the frequency of digging of helpers was negatively correlated with the amount of aggressive displays towards intruders when the dominants were present (supporting prediction 2, Fig. 2); this did not apply when the breeders were absent (*r*_*s *_= 0.38, *N *= 13; *P *= 0.20).

## Discussion

We found that the three measured behaviours of helpers in *N. pulcher *were consistent over time. There was a positive relationship between exploration behaviour and aggressive displays (a component of territory defence) and, when breeders were present, we found a negative relationship between territory maintenance and aggressive displays of helpers. These results suggest, that the behavioural correlations exhibited by brood care helpers of *N. pulcher *are components of behavioural syndromes. Furthermore, our results indicate that different behavioural traits may correspond to different types of helpers with regard to how they cooperate (i.e. territory maintenance or territory defence). Such 'cryptic task sharing' among helpers may only be detected when studying different helping behaviours repeatedly at an individual level [see also [31]].

Behavioural syndromes may either indicate constraints to the independent evolution of behaviours or suites of functionally integrated behaviours favoured by selection [12], i.e. coping strategies. Our results are in accordance with an adaptive nature of behavioural syndromes in helpers in *N. pulcher *because the detected correlations of behaviours correspond to the specific challenges individuals encounter when pursuing different life history strategies. Staying in the natal territory for a prolonged time period should correspond with a low inclination to be aggressive and to engage in risky exploration, but with a high tendency to engage in territory maintenance. In contrast, dispersing early should correspond to a high tendency to explore new habitats and to exhibit territory defence, but a low inclination to maintain the territory.

### Potential fitness consequences of behavioural syndromes in brood care helpers

Although we did not measure the fitness consequences of the detected behavioural combinations in this study, information on *N. pulcher *is available from the field and lab about fitness consequences of each of the measured behaviours. (a) Earlier studies suggest that exploration may facilitate between group dispersal to a nearby territory [28,29]. In case of expulsion from the group or group dissolution, explorative individuals may therefore have an advantage, as they can find shelter in the territory of another group. On the other hand, non-explorative individuals suffer a lower risk of predation [32]. (b) Experiments suggest that individuals staying in the home territory need to pay by engaging in helping behaviour [25,27,28,33,34]. Helping can therefore assure that helpers have access to a shelter [25,35] and it may provide the opportunity to inherit the territory, thereby conferring important fitness benefits [33]. However, helping also involves significant costs in that it is energetically expensive [36,37], and staying as a subordinate involves additional costs due to a strategic reduction of growth [28,35,38]. (c) Individuals aggressively defend their territory against unfamiliar intraspecific intruders [27,35], interspecific intruders and predators [32,35], which has been shown to have positive fitness effects [35]. However, aggression is low within groups and between familiar individuals of neighbouring groups [29]. Therefore, a general tendency to behave aggressively might have fitness reducing consequences, particularly when aggressiveness carries over to interactions within group members.

In summary, the available data suggest that each of the behaviours that is part of the behavioural syndromes we found, should have significant but contrasting fitness consequences when individuals indeed specialise into one of both options of staying or dispersing. The consistency in the behaviours and the correlations between the different types of behaviour may indicate strategic niche specialization [3,39] with regards to different life history trajectories: queuing for the breeding position in the home territory or dispersing to breed independently. Our data suggest, behavioural syndromes may affect how much and which type of help individual helpers in cooperative breeders will provide [31]. Future studies should help to clarify the respective fitness consequences of the detected behavioural correlations and how behavioural syndromes may affect the type and level of cooperative behaviour in cooperative breeders and other cooperative species [31,40-42].

### Factors influencing the correlations between behaviours

The negative relationship between digging and aggressive displays was only apparent in the presence of dominants, i.e. when subordinates have to pay for group membership [27]. This may suggest that the specialization becomes more expressed in the presence of dominants than when helpers dig and defend for self-serving purposes only.

Notably, aggressive displays against intruders significantly decreased when dominants were present compared to when they were absent. When the dominants are absent, the helpers might compensate for the missing participation in defence by absent group members, as has been shown in an earlier study [27].

Interestingly, aggressive displays were consistent within individuals, whereas overt attacks were not. This may be due to the different functions of both components of territory defence. While aggressive displays serve to threaten an opponent, overt attacks involve physical contact and demonstrate escalated aggression. Overt attacks might be more variable because they depend on the behaviour of the intruder whereas aggressive displays may rather reflect the 'intrinsic' aggressiveness of an individual.

The correlations between the behaviours were significant when analysing only female test scores and they remained so, also when analysing all individuals together, i.e. including the three males. However, the sample size, particularly for males, is small. Therefore, future studies are needed to clarify whether sex differences may influence the correlations among exploration, territory maintenance and territory defence among helpers.

## Conclusion

Our study provides evidence for the hypothesis that adaptive behavioural syndromes may result from a functional integration of different behavioural traits when individuals within a species adopt different strategies to cope with certain sets of ecological and social challenges.

## Methods

### Study species

*N. pulcher *is a cooperatively breeding cichlid fish [24,25,35,43] belonging to the substrate-breeding Lamprologini, and is endemic to Lake Tanganyika. It lives in family groups consisting of a breeding pair and on average five to ten helpers [29,43] of both sexes and different age classes [24]. Dominance depends on relative size and small helpers tend to engage more in territory maintenance and brood care, whereas large helpers engage more in territory defence [25,35]. Relatedness between group members is on average low and depends on their age, mainly because of frequent breeder replacement [44]. Groups defend small territories along the rocky sub-littoral zone and use holes and crevices for hiding and breeding.

### Test procedure

We tested immature individuals repeatedly for exploration behaviour, and for two types of helping behaviour that serve different social functions, i.e. territory maintenance (removing sand from the breeding shelter, i.e. 'digging') and territory defence (aggressive behaviour against a same sized conspecific intruder). To that end, we separated them from their original groups at an average standard length (SL) of 27.8 mm ± 1.6 min (mean ± SD; N = 13, min = 25 mm, max = 30 mm), i.e. they were not sexually mature at the onset of the experiment [25]. The fish originated all from 12 different breeding groups and one aggregation (a non-reproductive social group, where no breeding shelters were available). They were kept individually in 100 l tanks at conditions resembling those in Lake Tanganyika [25,35]. A clay flower pot halve served as shelter. The experiment was approved by the Swiss Federal Veterinary Office (Department of Economic affairs).

### (a) Exploration

Exploration tests were conducted with 12 individuals (9 females and 3 males) in a 400 l tank containing 10 numbered clay flower pot halves. The test fish was acclimatized for 15 min in a small plastic container with air supply that contained water from the test tank to allow it to adjust to the water quality of the test tank. Thereafter, the fish was released into a corner compartment created with an opaque partition that prevented the test fish from seeing the exploration tank, and was again allowed to acclimatize for 3 min. The test started by carefully removing the opaque partition creating the corner compartment. As a measure of exploration we recorded how many of the 10 available pots were explored during 15 min (fish entered the pot halve or approached it at a distance of 3 cm or less). To assure that the fish were exploring a novel environment we varied the exploration task by changing the side (left or right front side) where the test started and by varying the arrangement of the pot halves for each of the three tests of an individual. When seen from above, the distribution of the ten pot halves within the tank described either an M-shaped or a W-shaped arrangement (i.e. the arrangement was reversed). Half of the tested fish were subjected to the M-arrangement in the first test and to the W-arrangement in the second test whereas for the other half of the tested fish the sequence was reversed. In a third test all individuals were tested to explore a double W-shaped arrangement of the ten pot halves. In all three tests the distances between two adjacent pot halves were kept about equal. Between exploration tests 1 and 2 there was a period of 30 days, and between tests 2 and 3 a period of 65 days.

### (b) Helping

Digging and territory defence behaviour were tested in the presence and absence of dominants. For that purpose, we established a temporary group by adding two dominants (a small female and a large male, both larger than 45 mm SL) into the test helpers' tank. Translocated individuals usually establish and defend their territory against unfamiliar individuals soon after translocation when they have a substrate that serves as shelter and breeding substrate (here clay flower pot halves). All dominants originated from an aggregation tank. One additional male helper was used for these tests. During the course of the test for digging in the presence of breeders one male helper was expelled from the group (it kept hiding in a shelter) and therefore had to be removed. Hence, the sample sizes were *N *= 13 for territory defence and *N *= 12 for digging. The first test for helping behaviour was conducted 6 days after the second exploration test. After 4 days of acclimatisation we tested all helpers for territory defence behaviour and after three more days for digging behaviour. Subsequently, we removed the dominants from the groups and 7 days after the first test of each behaviour we repeated both tests in the absence of dominants.

### Territory defence (aggression)

We conducted the tests for territory defence against a conspecific intruder in the home tanks of the tested fish. 30 min before the test, we equalized the sand on the bottom of the tanks for optimal observation conditions and inserted a clear partition that separated the 100 l tank into two equal halves in order to separate the helper from the dominants and prevent interference of dominants. A second clear partition was inserted in the compartment of the tested helper to create a corner compartment for presentation of the intruder. Intruders presented during tests originated from an aggregation and were size matched to each of the tested fish. The test started when an intruder was released into the intruder presentation compartment. We recorded the frequency of overt attacks (ramming, biting, mouth fight) and restrained aggressive displays (fast frontal approach, head-down display, S-shaped bending, head jerking, opercula spreading, raising dorsal fin [25,35]) for a period of 10 minutes. We analysed the two classes of defence behaviours separately as they differ in intensity and may have different functions (aggressive displays are threat signals, whereas overt attacks intend body contact and physical restraint) [45].

### Territory maintenance (digging)

We covered the shelter (pot halve) in the compartment of the test helper with sand and allowed the fish to adjust to this potential disturbance for 10 minutes. Thereafter, we recorded the frequency of digging events (i.e. the test fish took sand into its mouth from inside the shelter, moved out of the shelter and spit out the sand) for a period of 10 minutes. Again, the dominants were separated from the test helpers with a clear partition in the first test, and had been completely removed before the second test.

### Data analysis

The repeatability coefficient for exploration behaviour was calculated [46]. Repeatability estimates (i.e. intraclass correlation coefficients) are commonly based on the variance components derived from a one-way ANOVA where r = s^2^_A_/(s^2 ^+ s^2^_A_), where s^2^_A _is the among-groups variance component and s^2 ^is the within group variance component. However, as our measurements of digging and territory defence behaviour involved different treatments (dominants present/not present), the repeatability of behaviours within individuals was calculated with the variance components obtained from a two-way ANOVA including individual as random factor and treatment as a fixed factor to correct for potential treatment effects. Repeatability coefficients were only calculated when individuals had a significant effect. To check for normality assumptions, the residuals of the analyses were tested for normality with Kolmogorov-Smirnov tests for goodness of fit, and the distributions were tested for deviations from homogeneity of variances with the Levene's test. Digging behaviour was ln (x+1) transformed to satisfy the assumption for homogeneity of variances.

To investigate the predicted relationships among the behaviours we calculated the average test scores for each repeatable behaviour (i.e. exploration (3 measurements), digging (2 measurements) and aggressive displays (2 measurements) and tested for the predicted positive correlation between exploration and territory defence, and the predicted negative correlation between these two behaviours and territory maintenance with help of Spearman rank correlation analyses. As we had a priori predictions for the expected correlations between the different behaviours we did not adjust the alpha-levels with a Bonferroni correction. However, all significant main correlations (i.e. between the three behaviours exploration, digging and aggressive defence (displays)) remain significant when adjusting the p-value for multiple comparisons.

## Competing interests

The author(s) declares that there are no competing interests.

## Authors' contributions

RB designed the experiments, carried them out, analysed the data and drafted the manuscript. MT participated in the design of the study and helped to develop the theoretical background and to draft the manuscript. Both authors read and approved the final manuscript.
